# Activation of TRPV3 channels in bladder cancer cells stimulates ATP release

**DOI:** 10.1016/j.molpha.2025.100096

**Published:** 2025-11-29

**Authors:** Jonas Janenz, Andrea Leipe, Nicole Urban, Michael Schaefer, Kerstin Hill

**Affiliations:** Rudolf-Boehm-Institute for Pharmacology and Toxicology, Leipzig, Germany

**Keywords:** Transient receptor potential vanilloid 3 channel, Bladder cancer cells, ATP release

## Abstract

Transient receptor potential vanilloid 3 (TRPV3) is a thermosensitive Ca^2+^-permeable ion channel that plays essential roles in epithelial barrier function. Although its expression and function have been well characterized in the skin and, to a lesser extent, in the gastrointestinal tract, its role in the urinary bladder has remained unexplored. In this study, TRPV3 was identified in human bladder cancer cell lines, and its functional activation was demonstrated, using a novel small-molecule agonist activator of TRPV3 channel 1 (AV3-1), discovered through medium-throughput screening. AV3-1 activated mouse and human TRPV3 channels with higher potency than known TRPV3 activators in Ca^2+^ assays and electrophysiological recordings. TRPV3 activation in the KU-19-19 bladder cancer cells stimulated ATP release, which was abolished by pharmacological TRPV3 blockade, confirming target specificity. Cholesterol supplementation further enhanced TRPV3 activity in KU-19-19 cells, a finding of potential relevance given the known dysregulation of cholesterol metabolism in bladder cancer. These results provide the first evidence of functional TRPV3 expression in bladder cancer cells and suggest that TRPV3 may contribute to Ca^2+^- and cholesterol-dependent signaling pathways. Collectively, these findings support further investigation of TRPV3 as a potential pharmacological target and exploratory biomarker in urothelial carcinoma.

**Significance Statement:**

TRPV3 is an ion channel mainly found in the skin. This study has identified the small molecule AV3-1 as a novel TRPV3 activator. Using AV3-1, this study demonstrates TRPV3 expression in bladder cancer cells. TRPV3 activation in these cells triggers ATP release, a signal potentially promoting cancer progression.

## Graphical abstract

**Figure:**
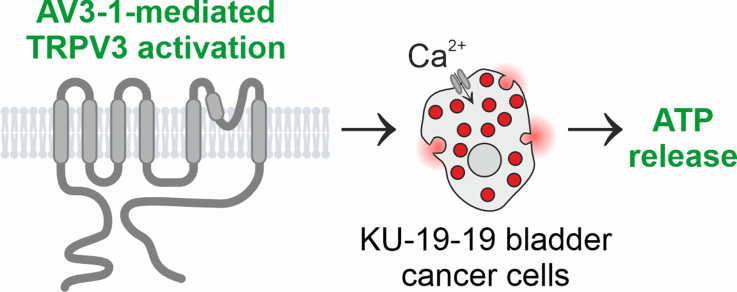

## 1 Introduction

Transient receptor potential vanilloid 3 (TRPV3) is a thermosensitive ion channel that has been shown to be foremost expressed in keratinocytes, where it plays a crucial role in the formation and the maintenance of the skin barrier.^1^, ^2^, ^3^, ^4^ Its dysfunction is associated with severe skin disorders, highlighting its potential as a therapeutic target in dermatological diseases.^5^, ^6^, ^7^

Beyond the skin, TRPV3 is expressed in several other epithelial tissues, including the distal colon,^8^^,^^9^ where its expression has been identified as a prognostic risk factor for the development of colon cancer.^10^ Consistently, colon carcinoma cells, such as the DLD-1 cell line, display high functional expression of TRPV3.^8^ Additional epithelial sites with reported TRPV3 expression include the bronchial epithelium,^11^ human larynx,^12^ corneal epithelial cells, and the oral mucosa.^13^ According to publicly available data sets^14^ (www.proteinatlas.org), low-level TRPV3 expression is also observed in the urinary bladder. However, functional data about TRPV3 in healthy bladder tissues or bladder carcinoma cells are lacking to date. Although the role of TRPV3 in bladder physiology and pathophysiology remains unexplored, other transient receptor potential (TRP) channels have been more extensively studied in both healthy urothelium and bladder cancer cells. For instance, TRPV4 is abundantly expressed in the urothelium, and its activation leads to the release of sensory mediators, such as ATP.^15^, ^16^, ^17^ Furthermore, functional expression of TRPV2 and TRPM7 has been detected in MBT-1 mouse bladder cancer cells, where these channels are proposed to act as negative regulators of tumor cell proliferation.^18^

As a thermosensitive ion channel, TRPV3 is activated by warm temperatures, with a threshold ranging from 31 to 39 °C.^4^^,^^19^ Beyond its thermal sensitivity, TRPV3 is modulated by various natural and synthetic compounds. One of the most widely used activators is 2-aminoethoxydiphenyl borate (2-APB), which is often employed to investigate TRPV3 function in cells that endogenously express the channel. However, caution is warranted because 2-APB activates or inhibits a variety of other TRP channels, making it difficult to clearly attribute the effects elicited by 2-APB solely to TRPV3.^20^, ^21^, ^22^, ^23^ Additionally, naturally occurring monoterpenes, such as camphor, carvacrol, eugenol, or citronellal,^24^^,^^25^ have been shown to act on TRPV3; however, high concentrations are required for robust TRPV3 activation, and they also lack specificity. In contrast, KS0365 demonstrates higher specificity for TRPV3, but it can induce nonspecific increases in intracellular Ca^2+^ at higher concentrations,^26^ and efficacy for the activation of the human orthologue is low.

Utilizing activator of TRPV3 channel 1 (AV3-1) as a novel pharmacological tool, we demonstrate here for the first time that TRPV3 is broadly expressed in bladder cancer cell lines. Moreover, our results establish AV3-1 as a robust and species-overarching activator suitable for exploring the functional roles of TRPV3-mediated Ca^2+^ signaling pathways in urothelial cancers. These findings not only provide novel insights into the molecular pharmacology of TRPV3 channels but also open new avenues for investigating their potential as therapeutic targets or biomarkers in bladder cancer.

## 2 Materials and methods

### 2.1 Chemicals and reagents

HEPES-buffered saline (HBS) consisted of 140 mM NaCl (Merck), 6 mM KCl (Carl Roth), 1 mM CaCl_2_, 10 mM HEPES (Carl Roth), and 5 mM glucose (Merck), adjusted to pH 7.4 with NaOH. In some experiments, HBS additionally contained 0.1% bovine serum albumin (BSA) (Sigma Aldrich). The Enamine Diversity Set compound library and AV3-1 were purchased from Enamine. AV3-1 was diluted in 100% DMSO to a stock solution of 100 mM and was subsequently diluted into whole-cell buffer for electrophysiological recordings or HBS at the desired working concentrations. 26E01 was synthesized in the laboratory of Oliver Thorn-Seshold.^8^ All other chemicals were purchased from Sigma Aldrich.

### 2.2 Cholesterol modification

Supplementation of the cellular cholesterol content in KU-19-19 cells was performed by adding cholesterol-loaded methyl-*β*-cyclodextrin (M*β*CD) to the cells. Cholesterol was dissolved in ethanol (20 mg/mL) by heating at 65 °C for 15 minutes. The cholesterol solution was then added dropwise to a 250 mM M*β*CD solution prepared in phosphate-buffered solution (PBS; Sigma Aldrich) at a 1:4 (v/v) ratio, yielding a stock containing 200 mM M*β*CD-cholesterol. KU-19-19 cells were incubated for 15 minutes at 37 °C in HBS containing 2.5 mM M*β*CD-cholesterol. As a control, KU-19-19 cells were incubated in HBS without M*β*CD-cholesterol supplement under the same conditions.

### 2.3 Cell culture

KU-19-19 (DSMZ no. ACC 395), CAL-29 (DSMZ no. ACC 515), 647-V (DSMZ no. ACC 414), RT-4 (DSMZ no. ACC 412), and RT-112 (DSMZ no. ACC 418) bladder cancer cell lines were obtained from the Leibniz Institute DSMZ (German Collection of Microorganisms and Cell Cultures). Human TRPV3 (NM_145068, purchased from OriGene) and mouse TRPV3 (NM_001371006), rat TRPV1, mouse TRPV2, mouse TRPV4, human TRPM2, human TRPM8, and human TRPA1 were stably transfected in HEK293 cells as described before.^27^ KU-19-19 and CAL-29 cells were maintained in RPMI medium (Sigma Aldrich), supplemented with 10% fetal calf serum (Capricorn Scientific), 1% l-glutamine (Sigma Aldrich), and 100 units/mL penicillin and 0.1 mg/mL streptomycin. HEK293 and HEK_TRPV3_ cells were cultured in Minimum Essential Medium (Sigma Aldrich) with 10% fetal calf serum, 1% L-glutamine, 100 units/mL penicillin, and 0.1 mg/mL streptomycin. Medium for cultivating HEK_TRPV3_ cells was supplemented with 0.5 mg/mL G418 (Capricorn Scientific).

### 2.4 Quantitative real-time polymerase chain reaction for hTRPV3

Total RNA was isolated from bladder cancer cell lines (CAL-29, 647-V, RT-112, RT-4, and KU-19-19) and the keratinocyte cell line HaCaT using standard extraction protocols. To quantify TRPV3 expression, quantitative real-time polymerase chain reaction (PCR) was carried out using the DyNAmo Flash SYBR Green qPCR Kit (Thermo Scientific, Cat. No. F416L) according to the manufacturer’s instructions. Specific primers for hTRPV3 were as follows: (1) forward (5′-GCC TAT GAA GGG CAG ACG GCG-3′) and (2) reverse (5′-TCC CGC GAG GTG ATG TCC GT-3′), each at a final concentration of 0.5 μM. The thermal cycling protocol consisted of an initial denaturation (95 °C for 7 minutes), followed by 44 cycles of denaturation (95 °C for 15 seconds), annealing (62 °C for 15 seconds), and extension (72 °C for 30 seconds), with a subsequent melt curve analysis (60–95 °C, 0.3 °C increment per second) to confirm amplification specificity. Threshold cycle (Ct) values were used to calculate *Δ*Ct values as Ct (TRPV3) − Ct (hActin). Relative expression levels were then determined using the *ΔΔ*Ct method, with HaCaT cells serving as the reference. Final expression values were expressed as 2ˆ-*ΔΔ*Ct.

### 2.5 Fluorometric [Ca^2+^]_i_ assays

All fluorometric Ca^2+^ assays were performed in HBS buffer at room temperature. The compounds of the Enamine Diversity Set were diluted in a buffer composed of 150 mM NaCl and 20 mM HEPES and added to the cells at a final concentration of 25 *μ*M (0.5% DMSO). For the primary compound screening and the generation of concentration-response analyses, a robotic liquid handling station (Freedom Evo 150; Tecan) with a built-in custom-made fluorescence plate imager was used. HEK_TRPV3_ and bladder cancer cell lines were harvested and loaded with 4 μM fluo-4/AM (Invitrogen; Fisher Scientific) in cell culture medium for 30 minutes at 37 °C. Cells were centrifuged at 1000 × g for 5 minutes, resuspended in HBS containing 0.1% BSA, and dispensed into black pigmented clear-bottom 384-well plates (Corning). Fluorescence was excited using a 470-nm light-emitting diode, and emitted light was imaged through a 515-nm–long pass filter with a cooled Zyla 5.5 camera (Andor). Fluorescence was recorded and processed under the control of the *μ*-Manager 2.0 gamma software (https://micro-manager.org.^28^). After recording baseline fluorescence, compounds were dispensed into the wells via the liquid handling device. Images were acquired every 1 second with an exposure time of 300 milliseconds. For further analysis, fluorescence signals were calculated for each well, corrected for background intensities and normalized to the initial signals. For fura-2-based microfluorometric single-cell Ca^2+^ assays, cells were seeded onto poly-L-lysine (PLL)–coated coverslips 24–48 hours before experiments. Cells were loaded with 4 μM fura-2-AM (Biomol) in HBS, containing 0.1% BSA for 30 minutes at 37 °C. After rinsing, coverslips were mounted in a bath chamber to the stage of an inverted Axiovert 100 microscope, equipped with a Fluar 10x/0.5 objective (Carl Zeiss). Fluorescence signals were excited with a fiber-coupled monochromator device (Polychrome V; Till Photonics) at 340, 358, and 380 nm and calibrated as described before.^29^ Activators and inhibitors were applied by continuous perfusion of extracellular solution using a gravity-driven system with a flow rate of 10 mL/min and a recording chamber with a volume of 0.4 mL.

### 2.6 Cell proliferation assay

To monitor a potential cytotoxicity of individual compounds, 3-(4,5-dimethylthiazol-2-yl)-2,5-diphenyltetrazolium bromide (MTT) assays were performed to assess cell viability and metabolic activity. Cells (5000 per/well) were seeded in PLL-coated 96-well plates and allowed to adhere overnight. After 24 hours, culture medium was exchanged for medium containing indicated drugs, and cells were cultured for another 24 hours. Following treatment with the compounds, 100 μL of medium containing 0.5 mg/mL MTT was added, and cells were incubated for additional 3 hours. The medium was removed, and 100 μL of DMSO was added to dissolve formazan crystals. Absorbance was measured at 560 nm, with a reference wavelength at 670 nm using a plate reader (Polarstar Omega; BMG Labtech). Cell viability was calculated relative to untreated control cells.

### 2.7 ATP assay

KU-19-19 cells (1 × 10^5^ per well) were plated in 24-well plates and cultured for 24 hours. Medium was removed, and wells were washed with PBS before adding 1 mL of drugs dissolved in PBS. After 30 minutes, 100 μL of the supernatant was transferred to a 96-well plate. ATP content was measured using the ATPlite luminescence ATP assay (Revvity, catalog number 6016943) following manufacturer’s instructions. Luminescence was recorded using a Polarstar Omega reader and normalized to control wells without addition of drugs.

### 2.8 Electrophysiological measurements

Whole-cell patch-clamp recordings were performed at room temperature using an Axopatch 700B amplifier connected to a Digidata 1440A digitizer under the control of the pCLAMP 10 software suite (Molecular Devices). Cells were plated onto PLL-coated glass coverslips 24–48 hours prior to recordings. Patch pipettes were pulled from borosilicate glass and had a resistance of 3–6 M*Ω*, when filled with intracellular solution, which contained 130 mM CsCl, 4 mM MgCl_2_, 10 mM HEPES, and 10 mM EGTA, pH of 7.2 adjusted with CsOH. The extracellular buffer consisted of 140 mM NaCl, 5 mM CsCl, 2 mM MgCl_2_, 1 mM CaCl_2_, and 10 mM HEPES, adjusted to pH 7.4 with NaOH. Recordings were performed with a liquid junction potential of +3.6 mV, which was not corrected. Voltage ramps ranging from −100 to 100 mV (500-millisecond duration) were applied at 1-second intervals. Series resistance was <15 M*Ω* and compensated by 70%. Only recordings with stable access resistance and minimal leak currents were included in the analysis. Activators and inhibitors were applied by continuous perfusion of extracellular solution using a gravity-driven system with a flow rate of 10 mL/min and a recording chamber with a volume of 0.4 mL.

### 2.9 Data and statistical analysis

During the experimental design, random groups of equal size were generated. Sample sizes were prespecified before data collection. Data are presented as box-and-whisker plots with individual data points (center line, mean; box, 25th–75th percentiles; whiskers, standard deviation [SD]) or means ± SDs. Concentration-response curves were fitted individually, and EC_50_ values were obtained for each experiment by applying a 4-parameter Hill equation: $y=E_{\min}+\frac{\left(E_{\max}-E_{\min}\right)x^{n}}{k^{n}+x^{n}}$ , where y is the response (F/F_0_), x is the concentration in μM, E_min_ is the minimum asymptote, E_max_ is the maximum asymptote, n is the Hill slope, and k is the EC_50._ For statistical analysis, EC_50_ values were log-transformed (pEC_50_ = -log_10_ (EC_50_[M])) to normalize the distribution. Mean values and SDs were calculated in log space, and for improved readability, the results are presented as back-transformed EC_50_ values. One-way ANOVA followed by Tukey post hoc test was employed for data analysis when normality distribution and variance homogeneity were confirmed by Shapiro-Wilk and Levene tests, respectively. In cases when homogeneity of variances was not met, the nonparametric Kruskal-Wallis ANOVA was applied, followed by Conover post hoc test for pairwise comparisons. Statistical analyses were performed using OriginPro 2023 software (OriginLab Corporation). Statistical significance was accepted at *P* < .05 (∗ = *P* < .05). This study was exploratory in nature. Consequently, *P* values are nominal and reported for descriptive purposes rather than formal hypothesis testing.

## 3 Results

**Fig. 1:**
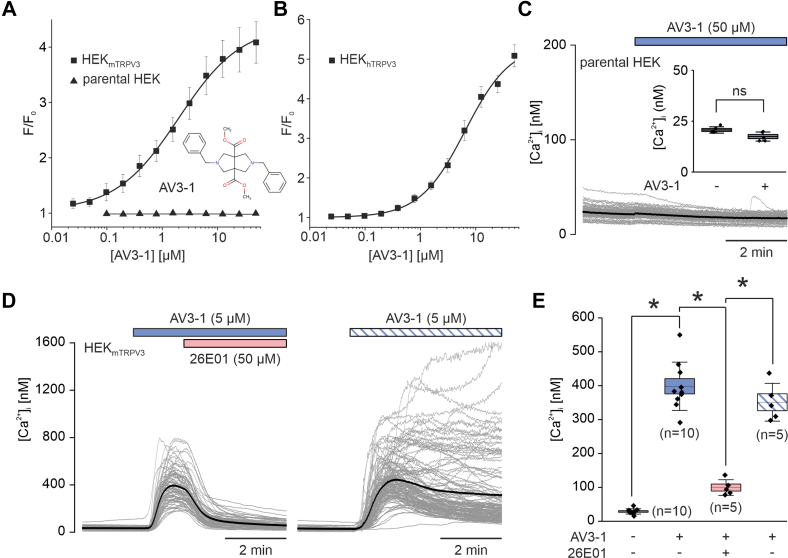
AV3-1 activated human and mouse TRPV3. (A, B) Concentration-response curves depicting TRPV3 activation by AV3-1 in HEK cells expressing either mouse TRPV3 (A, black squares) or human TRPV3 (B, black squares). Parental HEK cells lacking TRPV3 expression (A, black triangles) served as a negative control and showed no response to AV3-1. Fluorescence signals (F) were normalized to the fluorescence before compound addition (F_0_). Data are presented as mean ± SD from 5 independent experiments, with duplicates each. Chemical structure of AV3-1 is shown as in inset in (A). (C–E) Microfluorometric single-cell analysis of [Ca^2+^]_i_ in fura-2-loaded cells. Shown are representative traces of [Ca^2+^]ᵢ over time in parental HEK cells in response to application of 50 *μ*M AV3-1 (C) and in HEK cells stably expressing mouse TRPV3 (HEK_mTRPV3_), in response to application of 5 *μ*M AV3-1, followed by addition of the inhibitor 26E01 (50 *μ*M) (left panel) or without inhibitor (right panel) (D, E). Individual cell traces are shown in gray; the average trace is overlaid in black. Application periods are indicated by colored bars. (E) Quantification of peak [Ca^2+^]ᵢ responses from HEK_mTRPV3_ cells under the indicated treatment conditions. A baseline was recorded in all experiments, followed by AV3-1 application; in 50% of experiments, 26E01 was subsequently added to the cells. Because of this design, sample sizes differ across conditions; the exact *n* for each box is indicated in the figure. Quantification for parental HEK cells is shown as an inset in (C) (n = 5 independent experiments). Statistical significance was determined using one-way ANOVA with Tukey post hoc analysis; ∗*P* < .05. ns, not significant.

**Fig. 2:**
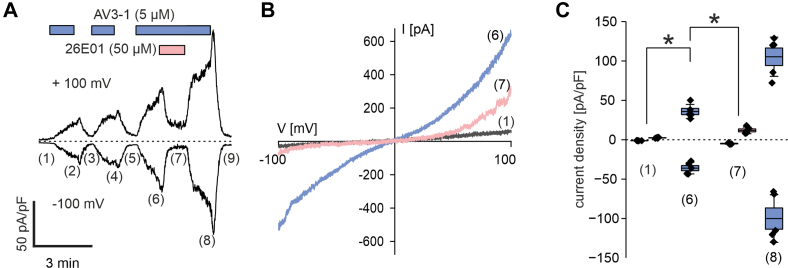
Electrophysiological analysis of AV3-1–activated TRPV3 currents in HEK_m__TRPV3_ cells. (A) Representative time course of a whole-cell patch-clamp recording obtained from a HEK_m__TRPV3_ cell, showing current responses to repeated applications of the TRPV3 agonist AV3-1 (5 *μ*M, blue bars, 1 minute each), followed by a washout period. The TRPV3 inhibitor 26E01 (50 *μ*M, red bar) was applied during the third AV3-1 stimulation. Current amplitudes were recorded using voltage ramps from −100 mV to +100 mV (500-millisecond duration) such as in B and expressed as current density (pA/pF) measured at +100 mV and −100 mV. The labels (1, 6–8) indicate time points for statistical analysis as shown in (C). (B) Representative current-voltage (I–V) curves from the same cell shown in (A), taken at baseline (1, black), at peak response to AV3-1 prior to 26E01 addition (6, blue), and during coapplication with 26E01 (7, red). (C) Quantification of current densities (pA/pF) at −100 mV (inward current) and +100 mV (outward current) at the time points (1, 6–8) as indicated in (A). Box plot shows data of n = 5 independent recordings. Statistical significance was determined using Kruskal-Wallis ANOVA followed by Conover test; ∗*P* < .05. Asterisks indicate significance for both outward and inward current components.

We recently identified KS0365 as a potent activator of TRPV3 channels.^26^ However, at higher concentrations (>10 μM), KS0365 exhibited nonspecific effects on HEK293 cells, limiting its suitability for use in native TRPV3-expressing systems. To discover novel TRPV3 modulators with improved selectivity profiles and, therefore, broader applicability, we performed a medium-throughput screen using the Enamine Drug Discovery Diversity Set, a compound library comprising 50,000 chemically diverse small molecules. Compounds were applied at a final concentration of 25 μM in the primary screen. After acute compound addition, primary hits were annotated when they induced increases in fluorescence signals. Fluorescence increases attributable to cytotoxic effects or to compound-intrinsic fluorescence were not pursued further. Candidate hits were validated by generating concentration-response curves in HEK293 cells expressing mouse TRPV3 and, in parallel, in parental HEK293 cells under identical assay conditions. Compounds were considered confirmed only if they produced reproducible responses in TRPV3-expressing cells with negligible activity in parental cells, consistent with on-target TRPV3 activation. This approach led to the identification of a novel TRPV3 activator, which we named AV3-1. AV3-1 (the structure is shown as an insert in Fig. 1A) exhibits drug-like properties as it adheres to the Lipinski rule of 5 with a molecular weight of 408 Da, a logP_O/W_ of 2.6, 0 H-bond donors, and 6 H-bond acceptors. AV3-1 robustly activated TRPV3 channels with EC_50_ values of 2.0 ± 1.4 μM for mouse TRPV3 (Fig. 1A) and 7.2 ± 1.4 μM for human TRPV3 (Fig. 1B) with no detectable off-target effects on parental HEK cells at concentrations up to 50 *μ*M (Fig. 1, A and C). These properties establish AV3-1 as the most potent activator of mouse and human TRPV3 channels identified to date. AV3-1–activated TRPV3 channels were inhibited by 26E01, a previously identified TRPV3-selective inhibitor (Fig. 1, D and E).^8^ To further characterize the biophysical properties of AV3-1–evoked TRPV3 currents, we performed whole-cell patch-clamp recordings in HEK_mTRPV3_ cells (Fig. 2, A–C). Application of 5 μM AV3-1 induced robust TRPV3-like currents that displayed a weak outward rectification and progressively increased upon repeated AV3-1 stimulation, which is a hallmark of TRPV3 channels.^4^^,^^30^ Coapplication of 50 μM 26E01 markedly suppressed currents, confirming the specificity of AV3-1. The effect of AV3-1 on TRPV3 was fully reversible as washout of AV3-1 reduced currents to almost baseline levels observed at the start of the recording.

Specificity tests across a range of closely and more distantly related TRP channels showed no effect on TRPV2, TRPV4, TRPM2, and TRPM8 channels at concentrations up to 50 μM and only a minor activation of TRPV1, making AV3-1 a promising tool to discriminate between different TRP channels endogenously expressed in native cells. However, AV3-1 also activates the promiscuous chemosensor TRPA1, which should be taken into account when interpreting experimental results in cell systems that express both TRPV3 and TRPA1 (Supplemental Fig. 1).

TRPV3 is expressed in a variety of epithelial tissues, including the skin, respiratory, and gastrointestinal tract and, to a lesser extent, the urinary bladder (www.proteinatlas.org).^14^ However, most available functional data on endogenous TRPV3 channels focus on keratinocytes with limited data available for colonic epithelial cells. To explore TRPV3 expression in urothelial cells, we quantified human TRPV3 mRNA levels across a panel of bladder cancer cell lines using quantitative real-time PCR. Expression levels were normalized to human *β*-actin (hActin) and further normalized to the HaCaT keratinocyte cell line (Supplemental Fig. 2). All bladder cancer cell lines tested exhibited detectable hTRPV3 mRNA expression. Among these, KU-19-19, CAL-29, and RT-4 cells showed the highest expression. These results confirm constitutive TRPV3 transcription in a range of bladder cancer cells, with expression levels exceeding those in HaCaT cells, a human keratinocyte cell line, in which TRPV3 expression has been well established.^27^

**Fig. 3:**
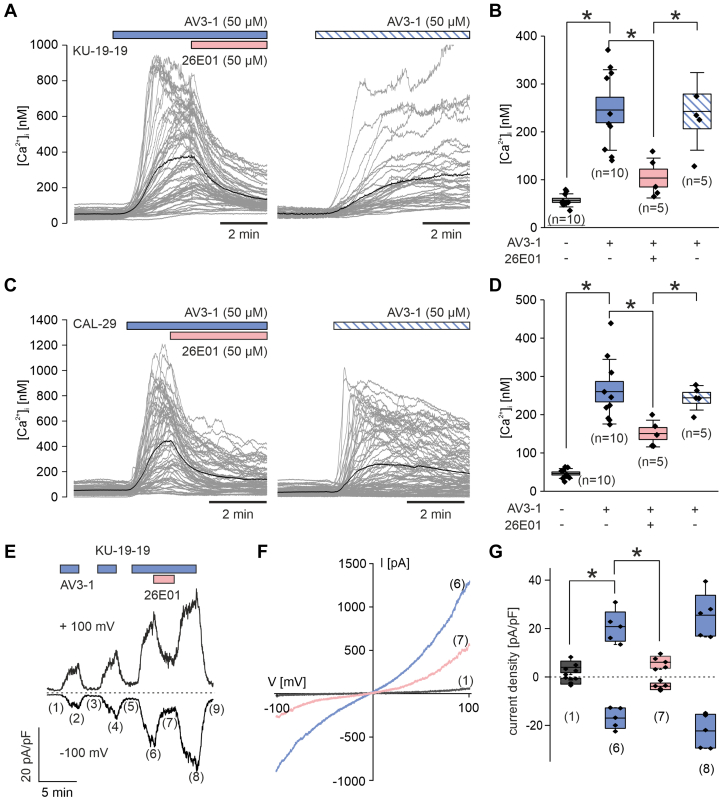
TRPV3 is functionally expressed in bladder cancer cells. (A–D) Activation of TRPV3 in KU-19-19 (A, B) and CAL-29 (C, D) bladder cancer cells by AV3-1, assessed by fura-2-based Ca^2+^ assays. The experimental layout was as in Figure 1, D and E. Shown are representative traces of [Ca^2+^]ᵢ over time in response to application of 50 *μ*M AV3-1, followed by addition of the inhibitor 26E01 (50 *μ*M) (left panel) or without inhibitor (right panel). Individual cell traces are shown in gray; the average trace is overlaid in black. Application periods are indicated by colored bars. (B, D) Quantification of peak [Ca^2+^]ᵢ responses from KU-19-19 (B) and CAL-29 (D) cells under the indicated treatment conditions. The exact *n* for each box is indicated in the figure. Statistical significance was determined using one-way ANOVA with Tukey post hoc analysis; ∗*P* < .05. (E–G) Electrophysiological whole-cell recordings performed on KU-19-19 cells. (E) Representative time course of a whole-cell patch-clamp recording obtained from a KU-19-19 cell, showing current responses to repeated applications of the TRPV3 agonist AV3-1 (50 *μ*M, blue bars, 1 minute each). The TRPV3 inhibitor 26E01 (50 *μ*M, red bar) was applied during the third AV3-1 stimulation. Current amplitudes were recorded using voltage ramps from −100 mV to +100 mV (500-millisecond duration) such as in F and expressed as current density (pA/pF) measured at +100 mV and −100 mV. The labels (1, 6–8) indicate time points for statistical analysis as shown in (G). (F) Representative current-voltage (I–V) curves from the same cell shown in (E), taken at baseline (1, black), at peak response to AV3-1 prior to 26E01 addition (6, blue), and during c-application with 26E01 (7, red). (G) Quantification of current densities (pA/pF) at −100 mV (inward current) and +100 mV (outward current) at the time points (1, 6–8) as indicated in (E). Box plot shows data of n = 5 independent recordings. Statistical significance was determined using Kruskal-Wallis ANOVA followed by Conover test; ∗*P* < .05. Asterisks indicate significance for both outward and inward current components.

To assess the functional activity of TRPV3 in the bladder cancer cells, we next measured intracellular Ca^2+^ changes following stimulation with the TRPV3 activator AV3-1 in KU-19-19 (Fig. 3, A and B) and CAL-29 (Fig. 3, C and D) cells because these cells lines exhibited a high TRPV3 expression. Fura-2-mediated Ca^2+^ assays revealed robust increases in [Ca^2+^]_i_ upon application of 50 *μ*M AV3-1 in both cell lines, which were statistically significant reduced by coapplication of the TRPV3 blocker 26E01. To further validate TRPV3 activity, we next performed electrophysiological whole-cell recordings similar to those conducted in HEK_m__TRPV3_ cells and confirmed that AV3-1 robustly and reproducibly activated TRPV3 currents in KU-19-19 cells (Fig. 3, E–G), which again were inhibited by acute addition of 26E01. The biophysical properties of the AV3-1–induced currents were consistent with those previously recorded in the HEK_m__TRPV3_ cell line. These findings demonstrate that TRPV3 channels are functionally active in bladder cancer cells and mediate Ca^2+^ influx upon stimulation.

**Fig. 4:**
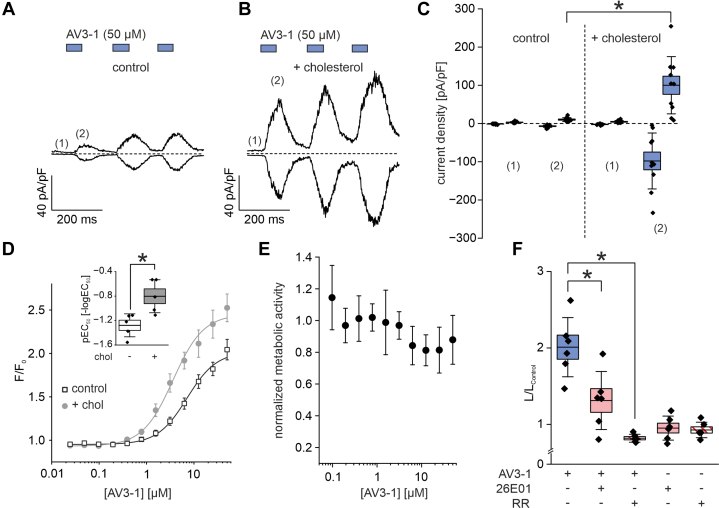
TRPV3 activation by AV3-1 was enhanced by cholesterol supplementation and causes ATP release in KU-19-19 bladder cancer cells. (A–C) Electrophysiological whole-cell recordings performed on KU-19-19 cells without (A) and with (B) cholesterol supplementation. (A, B) Representative time courses of whole-cell patch-clamp recordings obtained from a KU-19-19 cell, showing current responses to repeated applications of the TRPV3 agonist AV3-1 (50 *μ*M, blue bars, 1 minute each). Current amplitudes were recorded using voltage ramps from −100 mV to +100 mV (500-millisecond duration) and expressed as current density (pA/pF) measured at +100 mV and −100 mV. (C) Quantification of current densities (pA/pF) at −100 mV (inward current) and +100 mV (outward current) at the time points (1, 2) as indicated in (A, B). Box plot show data of n = 10 independent recordings. Statistical significance was determined using Kruskal-Wallis ANOVA followed by Conover test; ∗*P* < .05. Asterisks indicate significance for both outward and inward current components. (D) Concentration-response curves depicting TRPV3 activation in KU-19-19 cells without (open squares) or with (gray circles) cholesterol supplementation. Experiments were conducted as described in Figure 1, A and B, and depict means ± SD of 5 independent experiments. Inset: Box-and-whisker plots of AV3-1 potencies under control conditions (open box) and following cholesterol supplementation (gray box), derived from individual concentration-response fits and expressed as pEC_50_ (-log_10_(EC_50_ [M])). Each diamond represents an individual experiment (n = 5 per condition). Group differences were tested on pEC_50_ values using a two-tailed unpaired Student *t* test; ∗*P* < .05. (E) Concentration response analysis of AV3-1 in MTT assays with KU-19-19 cells depicting cell viability and proliferation after 24 hours of stimulation with AV3-1 at concentrations as indicated. Data represent means ± SD of n = 5 independent experiments. (F) Box plot quantification of extracellular ATP levels in KU-19-19 cells after stimulation with AV3-1 (50 μM, blue), measured by a luciferin-luciferase–based luminescence assay. Luminescence values are normalized to control values observed in wells containing only media (L/L control). Pretreatment with the TRPV3 antagonist 26E01 (50 *μ*M, red) or the nonselective TRP channel blocker ruthenium red (RR; 10 μM, red diagonal hatched fill) reduced AV3-1–evoked ATP release. Statistical analysis of n = 6 independent measurements was performed using one-way ANOVA with Tukey post hoc test; ∗*P* < .05. ns, not significant.

We previously reported that cholesterol supplementation sensitizes mouse TRPV3 channels to heat and 2-APB.^27^ Given that cholesterol is implicated in the progression of bladder cancer, with elevated cholesterol levels being associated with increased tumor occurrence, invasion, and metastasis,^31^ we investigated whether cholesterol similarly modulates human TRPV3 activity in bladder cancer cells. To this end, KU-19-19 cells were incubated with an M*β*CD-cholesterol complex prior to whole-cell patch-clamp recordings. Under cholesterol-loaded conditions, AV3-1 elicited substantially larger currents compared with untreated cells (Fig. 4, A–C), reproducing the sensitizing effect previously observed in heterologous expression systems expressing mouse TRPV3.^27^ To establish a concentration-response relationship for AV3-1–induced TRPV3 activation in KU-19-19 cells, we performed calcium assays. In cells not supplemented with cholesterol, the EC_50_ for AV3-1 was 19.0 ± 1.5 *μ*M (Fig. 4D, open square symbols), which is of a similar order of magnitude as values measured for heterologously expressed human TRPV3. Cholesterol supplementation increased the potency of AV3-1 threefold (EC_50_, 6.3 ± 1.9 *μ*M) (Fig. 4D, gray symbols) confirming the sensitizing effect of cholesterol-loading observed in the electrophysiological recordings.

To identify a concentration of AV3-1 that could be used for further assays without introducing cytotoxic effects, we performed MTT assays to assess cell viability and proliferation after incubation of the cells with different concentration of AV3-1. AV3-1 exhibited only minor toxicity at concentrations up to 50 *μ*M (Fig. 4E), indicating that the compound is well tolerated and suitable for extended application without compromising cell viability. To explore downstream effects of TRPV3 activation in bladder cancer cells, we next stimulated KU-19-19 with AV3-1 and quantified extracellular ATP levels. TRPV3 activation statistically significant increased ATP release compared with untreated control cells (Fig. 4F). To confirm that this ATP release was specifically mediated by TRPV3 activation, we applied the TRPV3-selective inhibitor 26E01 or the broad TRP channel blocker ruthenium red prior to stimulation with AV3-1. Both inhibitors reduced ATP levels in the supernatant, confirming that TRPV3 activation is required for AV3-1–induced ATP release (Fig. 4E). Altogether, we here present for the first time the presence and function of TRPV3 in bladder cancer cells.

## 4 Discussion

Several studies have identified distinct TRP channels to be involved in urothelial bladder function. TRPV1, for instance, is expressed in sensory fibers innervating the urinary tract.^32^ TRPV1-deficient mice exhibit abnormal bladder function characterized by frequent low-amplitude nonvoiding contractions.^33^ Conversely, increased TRPV1 expression has been linked to the pathophysiology of overactive bladder.^34^ Within the urothelium, TRPV1 activation has been reported to trigger the release of NO and ATP.^35^ However, the functional expression of TRPV1 in the urothelium of mouse and guinea pigs could not be confirmed by other groups,^36^^,^^37^ suggesting that TRPV1 expression in the bladder may be restricted to bladder afferent fibers and sensory neurons. Although TRPV1 has been relatively well characterized in bladder physiology and pathology, much less is known about other TRP channels. Functional expression of TRPV4, TRPV2,^38^ TRPM7,^36^ and TRPM8^36^ has been demonstrated in mouse urothelial cells. TRPV2 is highly expressed in T24/83 bladder cancer cells, where it is involved in promoting migration and invasion by increasing resting intracellular Ca^2+^ levels.^39^ Accordingly, TRPV2 expression is positively correlated with the advancement of bladder cancer.^38^^,^^40^ This is also the case for TRPM8, which shows markedly higher expression in cancerous than in noncancerous bladder tissues,^41^ and its enhanced expression is associated with shorter overall survival in bladder cancer patients.^42^ Similarly, TRPV3 has increasingly been recognized for its involvement in cancer progression. It is overexpressed in several malignancies, including non–small cell lung cancer, in which elevated TRPV3 expression is associated with reduced overall survival,^43^^,^^44^ and in breast cancer, where TRPV3 silencing inhibits cancer cell migration and proliferation.^45^ In renal clear cell carcinoma, high TRPV3 expression is correlated with a poor prognosis.^46^ Mechanistically, TRPV3 activation may promote cancer cell proliferation, migration, and survival by increasing intracellular calcium levels, which, in turn, activate downstream signaling pathways regulating cell cycle progression, such as the EGFR/AKT pathway.^45^

Here, we used the novel TRPV3 activator AV3-1, which we identified by screening a 50,000-compound library, to demonstrate that TRPV3 is functionally expressed in the KU-19-19 and CAL-29 bladder cancer cell lines. The currents induced by AV3-1 are clearly attributable to TRPV3 because AV3-1 does not activate other members of the TRP channel family known to be expressed in bladder cancer cells, including TRPV2, TRPV4, and TRPM8. Moreover, AV3-1–activated currents are blocked by the TRPV3 inhibitor 26E01^8^ and display distinct biophysical properties characteristic of TRPV3, including sensitization upon repetitive agonist application^3^^,^^4^ and sensitization by cholesterol supplementation.^27^ Based on these findings, we conclude that AV3-1 specifically activates TRPV3 channels in the KU-19-19 bladder cancer cells and is a suitable tool for further functional investigations into the role of TRPV3 in bladder cancer. Because TRPV3 is broadly expressed across all bladder cancer cell lines examined in this study, this points toward a general role of TRPV3 in bladder cancer. Future comparative analyses of TRPV3 expression in normal bladder urothelium and primary urothelial carcinoma will be essential to delineate a possible contribution of TRPV3 to bladder cancer. Given the Ca^2+^ permeability of TRPV3, the channel may act as a regulator of Ca^2+^-dependent signaling pathways in urothelial cancers. In the context of bladder cancer, Ca^2+^ signaling is a key regulator of tumor-promoting processes, such as proliferation, apoptosis, migration, and ATP release. Although AV3-1 did not affect proliferation of KU-19-19 cells in our experimental settings, we here demonstrate that treatment of AV3-1 leads to a TRPV3-dependent release of ATP. Extracellular ATP serves as a potent signaling molecule in the tumor microenvironment, influencing autocrine and paracrine signaling, immune modulation, and tumor progression (reviewed in the study by Yegutkin and Boison^47^). For example, ATP stimulates purinergic receptors on tumor and stromal cells, thereby promoting cell proliferation, survival, migration, and invasion.^48^^,^^49^ In addition, increased ATP levels within the tumor microenvironment drive epithelial-mesenchymal transition (EMT), which is a key step in cancer progression.^50^ Thus, TRPV3-mediated ATP release could contribute to establishing a protumorigenic milieu that favors bladder cancer progression and metastasis. This effect may be particularly relevant in light of the cholesterol-dependent TRPV3 potentiation observed in KU-19-19 cells. Cholesterol is a well established regulator of several TRP channels, including TRPV1,^51^ TRPV3,^27^ TRPM3,^52^ TRPC3,^53^ TRPM8,^54^ and TRPA1.^55^ In our study, cholesterol supplementation enhanced TRPV3 activity in bladder cancer cells, as evidenced by increased AV3-1 potency in calcium imaging assays and, even more pronounced, augmented TRPV3 currents in electrophysiological recordings. Given that cholesterol metabolism is frequently dysregulated in bladder cancer, where elevated total cholesterol levels have been associated with increased cancer risk and mortality,^31^^,^^56^ our findings suggest that TRPV3 may be a molecular participate in such cholesterol-driven signaling pathways that promote bladder cancer progression.

In conclusion, we provide the first evidence of functional TRPV3 expression in bladder cancer cells and show that its activation leads to Ca^2+^ influx and ATP release, both of which may contribute to tumor growth and metastasis. These findings raise the possibility that TRPV3 participates in Ca^2+^- and cholesterol-mediated signaling pathways in bladder cancer and may represent a potential biomarker or therapeutic target.

## Conflict of interest

The authors declare no conflicts of interest.
