# Supplementary material for: Activation of TRPV3 channels in bladder cancer cells stimulates ATP release
Source: Mol Pharmacol. 2025 Nov 29;108(1):100096. doi: 10.1016/j.molpha.2025.100096 (PMC12881674; doi:10.1016/j.molpha.2025.100096)
Supplement: Supplemental Material [file mmc1.docx]

**Activation of TRPV3 channels in bladder cancer cells stimulates ATP release**

Jonas Janenz, Andrea Leipe, Nicole Urban, Michael Schaefer, and Kerstin Hill

Rudolf-Boehm-Institute for Pharmacology and Toxicology, Härtelstrasse 16-18, 04107 Leipzig, Germany

**Corresponding author:**

Kerstin Hill: [kerstin.hill@medizin.uni-leipzig.de](mailto:kerstin.hill@medizin.uni-leipzig.de)

**Journal:**Molecular Pharmacology

**Supplemental Figure 1**

**
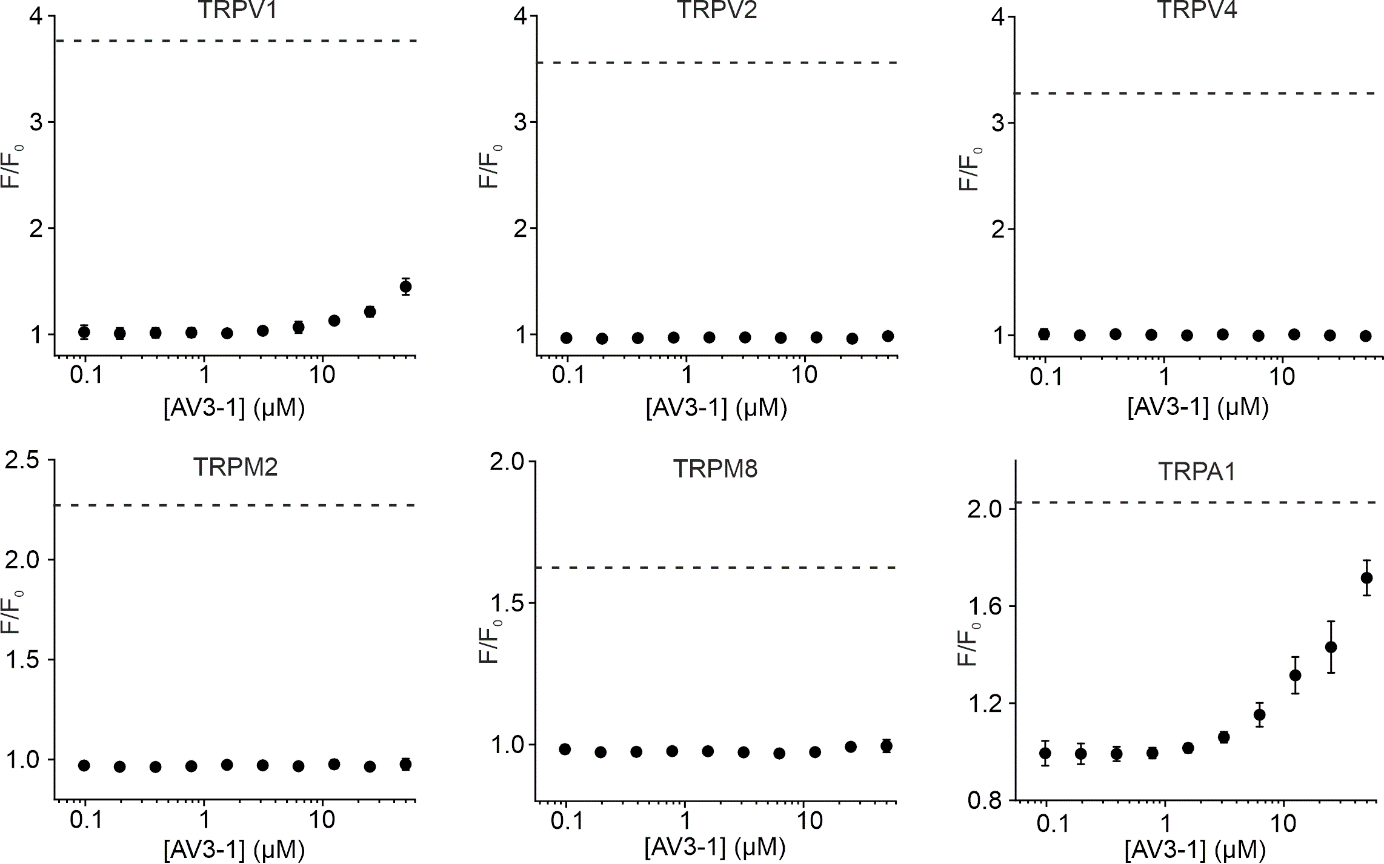
**

**Selectivity profile of AV3-1 across several TRP channels assessed by fluo-4-mediated Ca^2^**^+^ **assays.** Concentration-response curves depicting the response of HEK cells expressing different TRP channels to the addition of AV3-1. Fluorescence signals (F) were normalized to the fluorescence before compound addition (F_0_). Data are presented as mean ± SD from five independent experiments, with duplicates each. The channels tested are rat TRPV1 (TRPV1), mouse TRPV2 (TRPV2), mouse TRPV4 (TRPV4), human TRPM2 (TRPM2), human TRPM8 (TRPM8), and human TRPA1 (TRPA1). Fluorescence responses by reference activation are depicted as a dashed line in each diagram. Reference activators and concentrations were as follows: 2 µM capsaicin for TRPV1, 300 µM 2-APB for TRPV2, 1 µM GSK1016790A for TRPV4, 1 mM hydrogen peroxide for TRPM2, 300 µM menthol for TRPM8, and 100 µM AITC for TRPA1.

**Supplemental Figure 2**


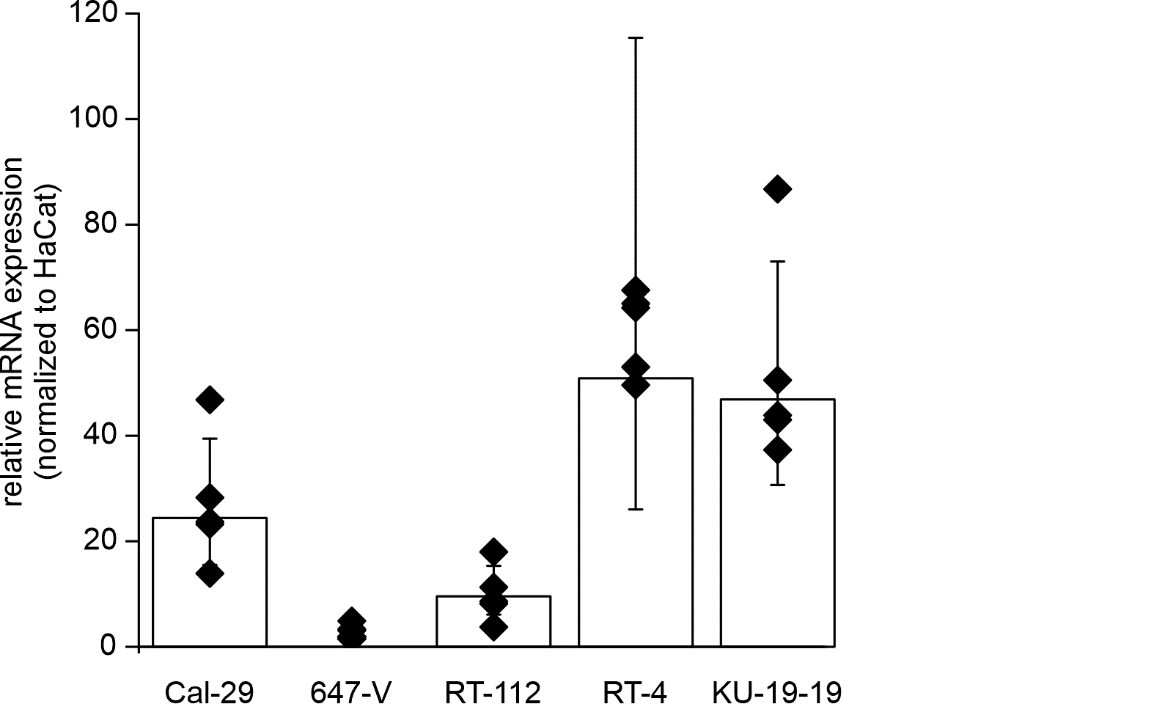


**TRPV3 is expressed in bladder cancer cells.**

Relative expression of hTRPV3 across several bladder cancer cell lines (CAL-29, 647-V, RT-112, RT-4, and KU-19-19). Gene expression was quantified by qRT-PCR and normalized to hActin using the ΔCt method. Relative expression levels were calculated as 2^-ΔΔCt, with HaCaT cells serving as the reference. Bars represent the mean ± SD of five biological replicates per cell line; individual data points indicate replicate measurements.
